# Reboxetine Improves Auditory Attention and Increases Norepinephrine Levels in the Auditory Cortex of Chronically Stressed Rats

**DOI:** 10.3389/fncir.2016.00108

**Published:** 2016-12-27

**Authors:** Catherine Pérez-Valenzuela, Macarena F. Gárate-Pérez, Ramón Sotomayor-Zárate, Paul H. Delano, Alexies Dagnino-Subiabre

**Affiliations:** ^1^Laboratory of Stress Neurobiology, Institute of Physiology, Center for Neurobiology and Brain Plasticity, Faculty of Sciences, Universidad de ValparaísoValparaíso, Chile; ^2^Laboratory of Neurochemistry and Neuropharmacology, Institute of Physiology, Center for Neurobiology and Brain Plasticity, Faculty of Sciences, Universidad de ValparaísoValparaíso, Chile; ^3^Program of Physiology and Biophysics, Institute of Biomedical Sciences (ICBM), Faculty of Medicine, Universidad de ChileSantiago, Chile; ^4^Otolaryngology Department, Clinical Hospital of the Universidad de ChileSantiago, Chile; ^5^Auditory and Cognition Center (AUCO)Santiago, Chile

**Keywords:** stress, norepinephrine, auditory cortex, attention

## Abstract

Chronic stress impairs auditory attention in rats and monoamines regulate neurotransmission in the primary auditory cortex (A1), a brain area that modulates auditory attention. In this context, we hypothesized that norepinephrine (NE) levels in A1 correlate with the auditory attention performance of chronically stressed rats. The first objective of this research was to evaluate whether chronic stress affects monoamines levels in A1. Male *Sprague–Dawley* rats were subjected to chronic stress (restraint stress) and monoamines levels were measured by high performance liquid chromatographer (HPLC)-electrochemical detection. Chronically stressed rats had lower levels of NE in A1 than did controls, while chronic stress did not affect serotonin (5-HT) and dopamine (DA) levels. The second aim was to determine the effects of reboxetine (a selective inhibitor of NE reuptake) on auditory attention and NE levels in A1. Rats were trained to discriminate between two tones of different frequencies in a two-alternative choice task (2-ACT), a behavioral paradigm to study auditory attention in rats. Trained animals that reached a performance of ≥80% correct trials in the 2-ACT were randomly assigned to control and stress experimental groups. To analyze the effects of chronic stress on the auditory task, trained rats of both groups were subjected to 50 2-ACT trials 1 day before and 1 day after of the chronic stress period. A difference score (DS) was determined by subtracting the number of correct trials after the chronic stress protocol from those before. An unexpected result was that vehicle-treated control rats and vehicle-treated chronically stressed rats had similar performances in the attentional task, suggesting that repeated injections with vehicle were stressful for control animals and deteriorated their auditory attention. In this regard, both auditory attention and NE levels in A1 were higher in chronically stressed rats treated with reboxetine than in vehicle-treated animals. These results indicate that NE has a key role in A1 and attention of stressed rats during tone discrimination.

## Introduction

Stress is a non-specific biological response of an organism to environment demands that affect their homeostasis. Consequently, stress allows them to restore homeostasis and adapt to environmental threats (Selye, 1936, 1956). Stressors are imminent or perceived challenges to homeostasis that activate the hypothalamus-pituitary-adrenal (HPA) axis, leading to the release of the steroids hormones from adrenal glands (cortisol in humans and corticosterone in rodents; Calabrese et al., 2007; Hennebelle et al., 2014).

Stress can be positive (eustress) when the stressors are mild, brief and controllable (Tafet and Bernardini, 2003). When the stressors are too intense and persistent (chronic stress), stress responses may become maladaptive, affecting brain structure and function (Tafet and Bernardini, 2003). In this sense, we have shown that the rat auditory system is sensitive to the effects of chronic stress (Dagnino-Subiabre, 2013). For instance, chronic stress and chronic corticosterone treatment induce dendritic atrophy in the central auditory pathways, such as the pyramidal neurons from primary auditory cortex (A1; Dagnino-Subiabre et al., 2005, 2009, 2012; Bose et al., 2010). These morphological alterations correlate with lower inhibitory synaptic efficacy in A1 compared to controls not exposed to chronic stress, as well as impairments in the auditory attention (Pérez et al., 2013).

In agreement with evidence obtained in rats, psychosocial stress and stress-related disorders (e.g., depression) in humans produce alterations in cognitive functions, such as attention (Gotlib and McCann, 1984; Mialet et al., 1996; Ottowitz et al., 2002; Kähkönen et al., 2007; Simoens et al., 2007; Kimble et al., 2010). Moreover, electroencephalographic recordings demonstrate that psychosocial stress in humans decreases general sound processing (mismatch negativity and N1/P2; Simoens et al., 2007).

Attention is a cognitive function that allows animals to select relevant stimulus in the environment and ignore irrelevant stimuli (Raz, 2004). The behavioral paradigm two-alternative choice task (2-ACT) is used to study auditory attention in rats (Otazu et al., 2009; Jaramillo and Zador, 2011; Znamenskiy and Zador, 2013; Xiong et al., 2015). In this paradigm, auditory attention is associated with increases in neural activity in A1, which is required to process information of attention (Hromádka and Zador, 2007; Xiong et al., 2015). As well, behavioral and electrophysiological studies have shown a significant positive association between the animal’s performances in the attentional task and increased activity in A1 (Xiong et al., 2015).

On the other hand, norepinephrine (NE) is released from the *locus coeruleus* nucleus (LC, located at the brainstem) during focused attention to relevant stimuli (Aston-Jones and Waterhouse, 2016). LC activity is modulated by eustress during attention tasks. For instance, eustress activates the corticotropin-releasing factor (CRF) neurons located in the LC (Merchenthaler et al., 1982; Palkovits et al., 1985) and consequently NE is released to brain areas involved in auditory attention, for example the auditory cortex (Salgado et al., 2012) and frontal cortex (Aston-Jones et al., 2000). Thus, NE-LC system enhances attention during the stress responses (Carli et al., 1983; De Martino et al., 2008). The A1 activity is strongly modulated by NE (Manunta and Edeline, 2000; Gaucher and Edeline, 2015), specifically pyramidal neurons located at layers II/III (Salgado et al., 2012). This neurotransmitter is released in A1 by projections from the LC. As a result, α_2_ or β adrenoceptors are activated, enhancing GABAergic synaptic transmission, while stimulation α_1_-adrenoceptors suppress inhibitory postsynaptic currents (IPSCs) in the A1 (Salgado et al., 2011a,b). Rats treated with an α_2_-adrenoceptor-antagonist drug show higher levels of attention than controls (Brown et al., 2012).

Together, these studies show that there is a relationship between LC-NE system, GABAergic transmission in A1 and auditory attention, which raise the question of whether chronic stress affects monoamine levels in the A1. Thus, the objectives of this study were to test whether repeated restraint stress affects: (1) NE, 5-HT and dopamine (DA) levels in the auditory cortex; and (2) the behavioral performance in an auditory attention task. We found that chronically stressed rats had lower levels of NE in A1 than controls, while 5-HT and DA levels were similar. We evaluated the effects of reboxetine on NE levels and on a performance in an auditory attention task. The main results were that control and chronically stressed rats treated with reboxetine had similar NE levels in the auditory cortex and comparable performance in the auditory attention task.

## Materials and Methods

### Ethics Statement

**Scheme 1:**
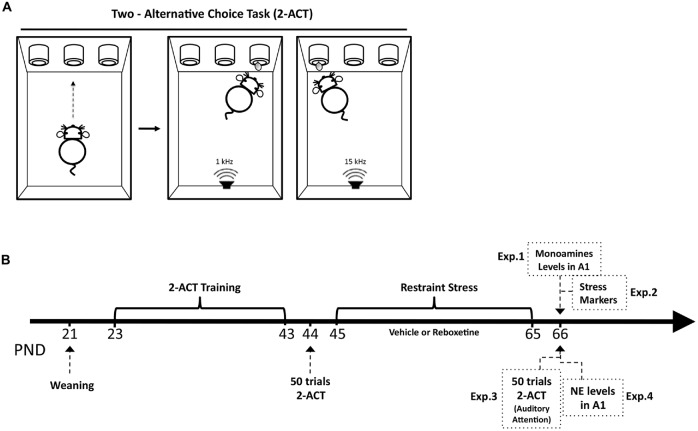
**Structure of the basic two-alternative choice task (2-ACT) and schematic drawing of the experimental design. (A)** 2-ACT is an auditory attention task; the trial initiates when the rat pokes its nose into the center port (*left*), which triggers the computer to randomly present one of two types of auditory stimuli, a high (15 kHz) or low (1 kHz) frequency tone (*right*). Rats were trained to respond with a left poke for high tones and a right poke for low tones; correct trials were rewarded with water. **(B)** The arrow represents the postnatal days of the animals (PND). After weaning, rats were trained in the 2-ACT for 20 days. Afterward, animals were randomly assigned to two experimental groups: control and stress. Trained rats were used in the Experiment 1 (Exp. 1) to determine monoamine levels in A1 1 day after chronic stress ended, and in Experiment 2 (Exp. 2) to analyze the stress markers. Experiment 3 (Exp. 3) analyzed the effects of chronic stress on auditory attention. Trained rats that were subjected to 50 2-ACT trials 1 day before and after the restraint stress protocol. A difference score (DS) was then determined by subtracting the correct trials after chronic stress (DS-CT) from those before. Experiment 4 (Exp. 4) analyzed NE levels in rats treated with reboxetine.

All procedures related to animal maintenance and experimentation were approved by the Institutional Animal Ethics Committee of the Faculty of Sciences Universidad de Valparaíso (Chile) and were in strict accordance with animal care standards outlined in National Institutes of Health (USA) guidelines. Efforts were made to minimize the number of animals used and their suffering. The antidepressant drug reboxetine at doses of 0.65 mg/kg (Pfizer, Pharmacia and Upjohn) or saline (vehicle, 0.9% NaCl) were intraperitoneal injected one time daily at 9:00–10:00 am for 21 days (Scheme 1B; Ampuero et al., 2015).

### Animals and Restraint Stress Protocol

Hundred sixty male *Sprague–Dawley* rats (control, *n* = 80, stress, *n* = 80) were used in all experiments of this study. Control animals, which were littermates of the stress-treated animals, were housed in separate rooms and cages, and not subjected to chronic stress. Restraint-stressed rats were placed in a plastic rat restrainer (6 cm diameter × 12 cm long and later 6 cm diameter × 20 cm long as the rats grew) in their home cages for 6 h daily, beginning at 10:00 h to 16:00 h for 21 consecutive days. Restraint occurred during the light phase of the light/night cycle. Animals were housed in groups of three per cage under a 12/12 light/dark cycle (lights on at 08:00 h), with *ad libitum* access to food (rat chow, Champion^®^, Santiago, Chile) and water in a temperature and humidity controlled room (20 ± 1°C, 55 ± 5%, respectively). Rats were weighed every day on a digital scale (Model WLC2/A1, Radwag, Poland).

After weaning (PND 23), 40 rats were trained for 3 weeks in a 2-ACT task, a behavioral task used to evaluate auditory attention in rodents (Scheme 1A). Trained rats were randomly distributed to two experimental groups: control, *n* = 20 and stress, *n* = 20.

## Two-Alternative Choice Task

### Stimuli Delivery and Apparatus

**Figure 1:**
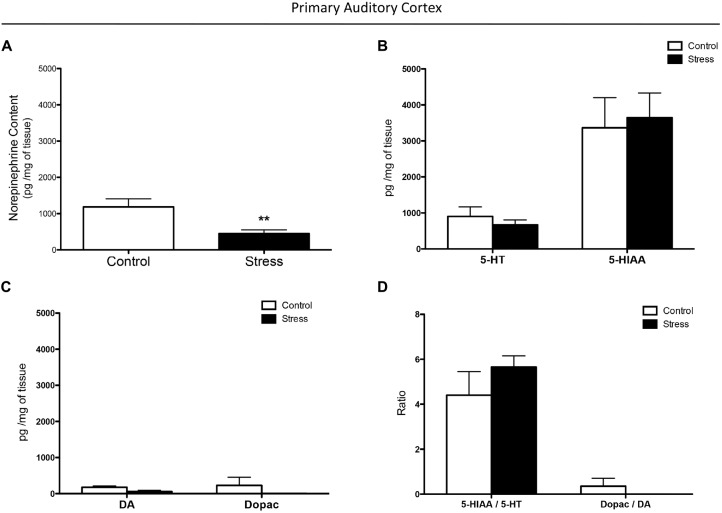
**Effect of chronic stress on monoamine levels in the auditory cortex. (A)** Chronic stress reduced norepinephrine (NE) concentration in the auditory cortex while it did not affect 5-HT **(B)** and dopamine (DA) **(C)** levels, and their metabolites 5-HIAA and dopac. Chronically stressed rats had comparable 5-HIAA/5-HT and dopac/DA ratios to those of controls **(D)**. **p < 0.01.

To determine auditory attention, the animals were trained to discriminate tones in a 2-ACT paradigm (Jaramillo and Zador, 2011; Xiong et al., 2015). Four modular rat operant chambers and accessories (LE1005, LE10022, LE100575, LE100560, Panlab S.L., Barcelona, Spain) were used in the attention task, each within a 67 cm^3^ × 67 cm^3^ × 67 cm^3^ sound-attenuating cubicle lined with 7.5 cm acoustic foam (Vroka S.A., Santiago, Chile). The operant chamber was illuminated to 200 lux (measured by a digital lux meter, Model # LX-1010B, Weafo Instrument Co., Shanghai, China) and the background noise level was 30 dB SPL. During training, the auditory stimuli were delivered through a speaker calibrated with a precision sound level meter (Model # 1100, Quest Technologies, Oconomowoc, WI, USA) to generate 70 dB SPL in the frequency range of 1–15 kHz at the position of the subject. The duration of the auditory stimuli was 0.1 s. The speaker was mounted in front of the three nose-poke, each connected with a liquid dispenser (Figure 1A). Operant modules were regulated by Packwin V1.2 software (Panlab S.L., Barcelona, Spain). All experiments were recorded with an IP camera (VIVOTEK, Sunnyvale, CA, USA) fixed above each operant chamber. Videos were acquired by Nuuo software (Nuuo, Taipei, Taiwan).

### Behavioral Task

Three days after weaning, male *Sprague–Dawley* rats (23 days old at the start of the experiment) were trained in the behavioral 2-ACT paradigm. Animals were water deprived over night under a protocol approved by the Institutional Animal Ethics Committee of the Faculty of Sciences, Universidad de Valparaíso, Chile. Afterward, the rat initiated a trial by inserting its nose into the central nose-poke of a three-port operant chamber (Scheme 1A), which triggered the computer to randomly present two types of acoustic stimuli: (1) a low frequency tone at 1 kHz; and (2) a high frequency tone at 15 kHz. The rats were trained to respond with right pokes for low frequency tones and left pokes for high-frequency tones. Correct trials were rewarded with water (Scheme 1A). All operant chambers were thoroughly cleaned with a 5% ethanol solution after each session. The control and chronically stressed groups were evaluated at the same time.

The 2-ACT training has three steps; in each of which it is possible to independently analyze learning, memory consolidation and auditory attention (Scheme 1B). In the first week of training, rats learned to respond with right pokes for low tones and left pokes for high tones (learning period). In the second week, rats were trained in 50 2-ACT trials until reaching 70% correct trials. At the end of this week the rats’ memory related to 2-ACT was consolidated (memory consolidation period). Beginning of the third week, the rats recalled the task and improved their auditory attention increasing correct responses to over 80% of trials (auditory attention period; Pérez et al., 2013).

### Experimental Design

Scheme 1B shows a schematic drawing of the experimental design used in this study. Table 1 shows the number of rats used in each experiment. Experiment 1 analyzed the effect of repeated restraint stress on monoamine levels in A1 (control, *n* = 6, stress, *n* = 6). Experiment 2 analyzed the effect of reboxetine on body weight gain and locomotor activity of rats after the chronic stress protocol ended (control: vehicle, *n* = 9, reboxetine, *n* = 9; stress: vehicle, *n* = 9, reboxetine, *n* = 9). The stress levels after the chronic stress period were also evaluated. The most conventional method to determine if the animals are chronically stressed is to measure plasma levels of the stress hormone corticosterone. Animals were exposed to a new stressor (forced swim) and corticosterone plasma levels were quantified before and after acute swim exposure (Unstimulated, control: vehicle, *n* = 6, reboxetine, *n* = 6; stress: vehicle, *n* = 6, reboxetine, *n* = 6. Acute swim, control: vehicle, *n* = 6, reboxetine, *n* = 6; stress: vehicle, *n* = 6, reboxetine, *n* = 6). In Experiment 3, we studied the effect of reboxetine on auditory attention of chronically stressed rats. After 3 weeks of training, rats that reached a correct performance of over 80% in 50 trials were included in this experiment. The animals were randomly assigned to two groups: control: vehicle, *n* = 10, reboxetine, *n* = 10 and stress: vehicle, *n* = 10, reboxetine, *n* = 10, and 1 day before and after of the chronic stress protocol, the rats were subjected to 50 trials of 2-ACT (Scheme 1B). Only in the Experiment 3 trained animals were used in the 2-ACT. Experiment 4 evaluated whether reboxetine affects the NE levels in the auditory cortex of chronically stressed rats (control: vehicle, *n* = 6, reboxetine, *n* = 6; stress: vehicle, *n* = 6, reboxetine, *n* = 6).

**Table 1:** **Number of rats used in each experiment**.

Experiment	Description	Experimental group				Total
1	Monoamine levels in Al	Control		Stress		
			
		*n* = 6		*n* = 6		*n* = 12
			
2	Stress markers	**Vehicle**	**Reboxetine**	**Vehicle**	**Reboxetine**	
	Body weight and locomotor activity	*n* = 9	*n* = 9	*n* = 9	*n* = 9	*n* = 36
	Corticosterone levels in unstimulated rats	*n* = 6	*n* = 6	*n* = 6	*n* = 6	*n* = 24
	Corticosterone levels after acute swim	*n* = 6	*n* = 6	*n* = 6	*n* = 6	*n* = 24
3	Auditory attention	*n* = 10	*n* = 10	*n* = 10	*n* = 10	*n* = 40
4	NE levels in Al	*n* = 6	*n* = 6	*n* = 6	*n* = 6	*n* = 24

**Total**						*n = 160*

## Experiment 1

### Monoamine Contents in the Auditory Cortex

After completion of the chronic stress protocol each rat was decapitated under deep anesthesia with isoflurane. The brain was removed quickly and the auditory cortex was microdissected at 4°C according to Hébert et al. (1987) and Basura et al. (2008). The tissue was collected in 400 μL of 0.2 N perchloric acid and then homogenized in a glass-glass homogenizer. The homogenate was centrifuged at 12,000 g for 15 min at 4°C (Model Z233MK-2, Hermle LaborTechnik GmbH, Germany) and the supernatant was injected into a high performance liquid chromatographer (HPLC) coupled to electrochemical detection, to measure NE, 5-HT and DA content. The contents of all monoamines were expressed as picograms per milligram of tissue. Ten microliters of each supernatant were injected into the HPLC system with the following setting: a isocratic pump (Model PU-2080 Plus, Jasco Co. Ltd., Tokyo, Japan), a UniJet microbore column (MF-8912, BAS, West Lafayette, IN, USA) and an amperometric detector (set at 650 mV, 0.5 nA; Model LC-4C, BAS, West Lafayette, IN, USA). The mobile phase, containing 0.05 M NaH_2_PO_4_, 1.0 mM 1-octanesulfonic acid, 0.27 mM EDTA, 1.0% (v/v) tetrahydrofuran and 4.0% (v/v) CH3CN (pH adjusted to 2.6) was pumped at a flow rate of 100 μL/min. Neurotransmitter levels were assessed by comparing the respective peak area and elution time of the sample with a reference standard and the quantification was performed using a calibration curve for each neurotransmitter (Program ChromPass, Jasco Co. Ltd., Tokyo, Japan). Under these experimental conditions, retention times were 3.4 min for NE, 7.1 min for DA and 16.0 min for 5-HT. Standards, EDTA and 1-octanesulfonic acid, were purchased from Sigma-Aldrich, Inc. (St. Louis, MO, USA), and all other reagents were of analytical grade. The detection limit for DA, Dopac, 5-HT, 5-HIAA and NE was 0.1 pg/mg of tissue.

## Experiment 2

### Stress Markers

#### Body Weight Gain

To analyze the effects of the stress procedure, we measured the percentage gain in body weight at the beginning and end of the experimental period (net change in weight after experiment ×100/weight at the beginning of the experiment).

#### Determination of Plasma Corticosterone

We used a separate set of rats to determine the corticosterone concentration in plasma to avoid the stress of blood collection affecting behavioral or HPLC experiments. A set of rats (control, *n* = 6, stress, *n* = 6) was subjected to 60 s of forced swim at 11:00 h, after which the animals were transferred to a heated holding cage for 10 min. Afterward, the animals were transported to a separate room (time used approximately 10 s), quickly anesthetized with isoflurane (time used approximately 5 s) and immediately sacrificed via decapitation under deep anesthesia to collect blood. Animals were not exposed to other decapitated animals before deep anesthesia. Another set of rats (control, *n* = 6, stress, *n* = 6) was not disturbed and sacrificed at 11:11 h under deep anesthesia. The forced swim consisted of a plastic beaker (25 cm in diameter, 46 cm deep) containing 30 cm of water (19 ± 1°C). Blood (1 mL) was collected in heparinized microcapillary tubes and centrifuged (Model # MiniSpin Plus; Eppendorf AG, Hamburg, Germany) at 10,000 rpm for 10 min to obtain plasma and then stored at −70°C. Total corticosterone was determined by an Enzyme Immunoassy kit (Corticosterone BioAssay^TM^, Catalog. # C7903-30) purchased from US Biological (Swampscott, MA, USA). Optical density values were measured at 450 nm using a micro-plate reader (Tecan GENios^TM^, Tecan Group Ltd., Switzerland). Samples were diluted 1:10 and then processed in duplicate and averaged final values were represented as ng/mL.

### Locomotor Activity

#### Open Field Test

Locomotor activity was evaluated in the open field test. All animals were naive to the test situations. The behavioral test was carried out from 10:00 h to 14:00 h. in the test room. The activity of each rat was recorded by IP cameras fixed above the behavioral apparatus and connected to a computer in another room. Videos were acquired by Nuuo software (Nuuo, Taipei, Taiwan). Behavioral tests were conducted in a soundproof and temperature-controlled (21 ± 1°C) room. Each rat was placed in the center of a black Plexiglass cage (70 cm × 70 cm × 40 cm) for 5 min. The background noise level in the open field was 40 dB SPL (Precision sound level meter, Model # 1100, Quest Technologies, Oconomowoc, WI, USA) and the arena was illuminated to 300 lux (measured by a digital lux meter, Model # LX-1010B, Weafo Instrument Co., Shanghai, China). Total distance traveled was analyzed from video recordings using ANY-maze video tracking system (Stoelting Co., Wood Dale, IL, USA). All mazes were wiped thoroughly clean with a 5% ethanol solution after each trial. In all experiments, animals from control and stress experimental groups were evaluated at the same time.

## Experiment 3

### The Effects of Reboxetine on Auditory Attention

Trained rats were subjected to 50 trials of 2-ACT 1 day before and after the restraint-stress protocol (Scheme 1B). To analyze the effects of reboxetine on the behavioral performance of chronically stressed rats in the auditory attention task, a DS was measured by subtracting the number of correct trials determined before chronic stress (DS-CT) from the number of correct trials obtained in the 50 trials after of the chronic stress period. Positive values for the DS-CT were associated with an increase in correct trials after the chronic stress period compared to correct trials before restraint stress. The number of correct trials was measured using the Packwin software.

## Experiment 4

### The Effects of Reboxetine Effects on NE Content in the Auditory Cortex

After completion of the restraint-stress protocol each rat treated with vehicle or reboxetine was decapitated under deep anesthesia with isoflurane. NE content in the auditory cortex was determined as shown in the description of Experiment 1.

### Statistical Analysis

#### Contents of Monoamines and Metabolites in the Auditory Cortex

NE concentration in A1 was analyzed using the non-parametric Mann-Whitney U test. The results shown in Figures 1B–D were analyzed by a two-way ANOVA. The independent variables were experimental groups (control and stress) and neurotransmitter and metabolite contents (5-HT, 5-HIAA, DA, and dopac) in Figures 1B,C, and the ratio (5-HIIAA/5-HT and dopac/DA) in Figure 1D. The dependent variables were pg/mg of tissue and the ratio.

#### Effects of Reboxetine on Body Weight Gain, Open Field Test and NE Content

Results were analyzed by a two-way ANOVA. The independent variables were treatments (vehicle and reboxetine) and stress (control and stress). The dependent variables were body weight (body weight gain), total distance traveled (locomotor activity), and pg/mg of tissue (NE content).

#### Corticosterone Levels

Corticosterone levels were analyzed by a 3 × 2 factorial ANOVA. The dependent variable was serum corticosterone levels and the independent variables were restraint stress (control and stress), treatments (vehicle and reboxetine), and acute stress (without stimulation and acute swim).

#### Auditory Attention

The performance of the animals in the 50 trials were analyzed by a two-way repeated-measure ANOVA (DS [groups (vehicle, reboxetine) × trials (1–50)]).

Bonferroni *post hoc* test was used for all experiments for multiple comparisons.

Data are represented by mean ± SEM. A probability level of 0.05 or less was accepted as significant. The asterisk (*) indicates significant differences relative to control animals.

## Results

### Experiment 1

#### Effects of Chronic Stress on Monoamine Contents in the Auditory Cortex

Chronically stressed rats had lower levels of NE in the auditory cortex than did control animals (*p* < 0.01; Figure 1A). As shown in Figures 1B–D a two-way-ANOVA revealed that restraint stress did not affect 5-HT and 5-HIAA concentrations (*F*_(1,10)_ = 0.0013, *p* = 0.9718), DA and dopac concentrations (*F*_(1,10)_ = 2.194, *p* = 0.1693), and the ratios of 5-HIAA/5-HT and dopac/DA (*F*_(1,10)_ = 0.3706, *p* = 0.5562). In both control and chronically stressed rats, the 5-HIAA concentrations were significantly higher than 5-HT levels (*F*_(1,10)_ = 37.63, *p* < 0.001; Figure 1B), while the 5-HIAA/5-HT ratio was higher than the dopac/DA ratio (*F*_(1,10)_ = 62.55, *p* < 0.0001; Figure 1D).

### Experiment 2

#### Stress Markers and Locomotor Activity

**Figure 2:**
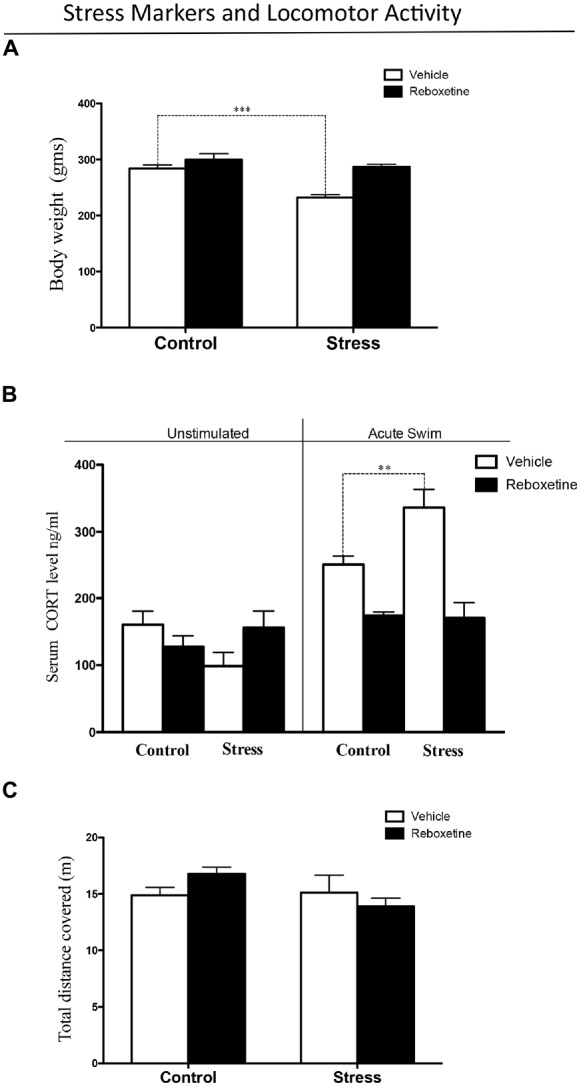
**Stress levels after 21 days of restraint stress paradigm. (A)** Chronic stress decreased body weight gain, while reboxetine prevented this effect. **(B)** After acute swim, serum corticosterone levels were significantly higher in chronically stressed rats treated with vehicle than in control animals (black bars), while reboxetine prevented this effect (black bars). **(C)** Reboxetine did not affect the locomotor activity of animals in the experimental groups. **p < 0.01, ***p < 0.001.

To determine whether our chronic stress protocol was effective in triggering stress response, we measured locomotor activity and stress markers, including the corticosterone level and body weight gain. There was a significant effect of the treatment-stress interaction on body weight gain (*F*_(1,16)_ = 6.803, *p* < 0.05). The *post hoc* test showed that chronically stressed rats treated with vehicle had significantly lower body weight gain than control rats (stress: 232.0 ± 5.6 g, control: 284.2 ± 6.4 g; *p* < 0.001; Figure 2A).

Figure 2B shows the level of circulating corticosterone in rats subjected to a 60 s forced swim and in animals that were not disturbed. Following acute swimming, both stress and treatment altered corticosterone levels (*F*_(3,20)_ = 4.644, *p* < 0.05). There was a significant effect of the treatment-stress interaction on corticosterone levels (*F*_(3,20)_ = 14.26, *p* < 0.001; Figure 2B). The *post hoc* test showed that vehicle-treated controls and chronically stressed rats that were exposed to acute swim had higher corticosterone levels than those were left undisturbed (*p* < 0.01). Following acute swimming, animals from the stress group had significantly higher corticosterone levels than did control rats (*p* < 0.01). Control rats treated with reboxetine that were subjected to acute swimming had lower levels of corticosterone than control rats treated with vehicle (*p* < 0.05; Figure 2B). In addition, chronically stressed rats treated with reboxetine that were subjected to acute swimming had lower levels of corticosterone than chronically stressed rats treated with vehicle (*p* < 0.001; Figure 2B). There was no difference between control and chronically stressed rats treated with reboxetine after acute swimming (*p* > 0.05; Figure 2B).

Neither restraint stress nor reboxetine affected locomotor activity. There was no main effect of restraint stress (*F*_(1,16)_ = 2.661, *p* = 0.1224) or reboxetine (*F*_(1,10)_ = 0.090, *p* = 0.7682) on the total distance traveled in the open field test (Figure 2C).

### Experiment 3

#### Effects of Reboxetine on Auditory Attention

**Figure 3:**
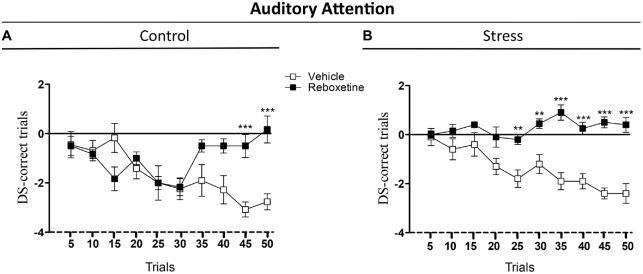
**Influence of reboxetine on the auditory attention of chronically stressed rats.** The difference score of correct trials (DS-CTs) of control **(A)** and chronically stressed rats treated with reboxetine **(B)** were significantly higher than those of vehicle-treated rats. **p < 0.01, ***p < 0.001.

The aim of this experiment was to evaluate the effect of the reboxetine on the auditory attention behavior of control and chronically stressed rats that were trained in the attentional task, an auditory frequency discrimination task used to study attention in rats (Figure 3). Reboxetine did not affect the DS-CT of control animals in the first 40 trials of the 2-ACT (Figure 3A). However, after the 40th trial, the DS-CT of reboxetine-treated rats was higher than that of vehicle-treated control rats (Figure 3A). There was a significant interaction between treatment and trial (*F*_(9,162)_ = 6.214, *p* < 0.001), while the effect of treatment was significantly altered (*F*_(9,18)_ = 5.189, *p* < 0.05). The DS-CT of the chronically stressed rats treated with reboxetine was higher than that of vehicle-treated rats in the trial 20 of 2-ACT (Figure 3B). Interactions between treatment and trial, and the effect of treatment increased significantly (Interaction: *F*_(9,162)_ = 4.678, *p* < 0.001); (Treatment: *F*_(9,18)_ = 165.3, *p* < 0.001; Figure 3B). These results demonstrate that reboxetine improved the behavioral performance of chronically stressed rats after 20th trial during the auditory attention task.

### Experiment 4

#### NE Concentration in the Auditory Cortex of Rats Treated with Reboxetine

**Figure 4:**
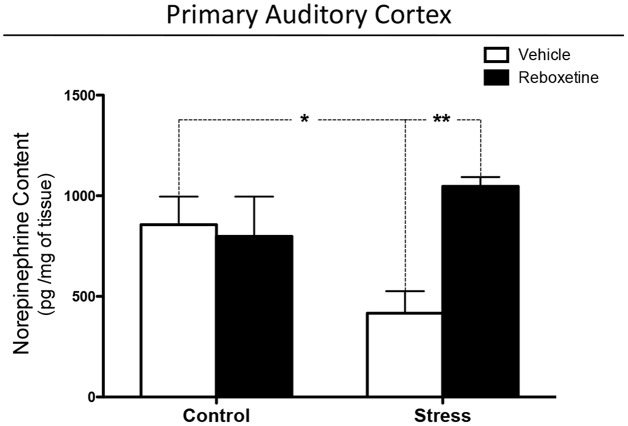
**Effect of reboxetine on NE levels in primary auditory cortex (A1).** Reboxetine increased NE in A1 of chronically stressed rats. The asterisk (*) indicates significant difference relative to control animals. **p < 0.01.

Chronically stressed rats treated with vehicle had lower levels of NE in the auditory cortex than did control animals (*p* < 0.05; Figure 4). Reboxetine prevented this effect, as chronically stressed rats that were treated with reboxetine had higher levels of NE than did chronically stressed animals treated with vehicle (*p* < 0.01; Figure 4). Interactions between treatment and chronic stress, and the effect of treatment increased significantly (Interaction: *F*_(1,20)_ = 6.498, *p* < 0.05); (Treatment: *F*_(1,20)_ = 4.482, *p* < 0.05; Figure 4).

## Discussion

In this study we found that chronic stress specifically decreased NE concentrations in A1, which could be explained in part by the relationship between the HPA axis and LC-NE system. It is possible that in our experiments chronic stress had decreased LC activity and NE release in the brain. This idea is supported by the study of Isingrini et al. (2016), who found that mice exposed to repeated social defeat stress, a chronic stress protocol, have lower LC activity and decreases NE levels in the ventral tegmental area (VTA) than compared to unstressed mice. It was also interesting that optogenetic stimulation of LC-NE fibers in the VTA reversed the stress phenotype (Isingrini et al., 2016). In this sense, the study of Pacák et al. (1993), shows that repeated immobilization stress decreases extracellular levels of NE and 3-Methoxy-4-hydroxyphenylglycol (MHPG, the main NE metabolite) in the central nucleus of the amygdala, suggesting that chronic stress decreases noradrenergic turnover but not the synthesis of NE in this brain area. The study of Sakaguchi and Nakamura (1990), demonstrates that chronic restraint stress decreases LC neuronal activity. Taken together these evidences suggest that there is a lower activity of the LC-NE system in chronically stressed rats than in control animals.

Having demonstrated that chronic stress decreases NE levels in A1, we determined if reboxetine affects auditory attention in chronically stressed rats. Firstly, we determined if the applied chronic stress procedure was effective in triggering stress responses in the animals treated with vehicle or reboxetine. The rats subjected to restraint stress and treated with vehicle had lower body weight gain than did controls injected with vehicle (Figure 2A). This alteration has been generated by chronic stress in different animal models and can be explained by increased glutamatergic activity in the brain, which induces chronic stress in the limbic system. One effect of this neurophysiological alteration is increased energy demand provided by lipid catabolism, which in turn results in lower weight gain, as can be observed in Figure 2A. This correlates with higher plasma corticosterone concentrations in chronically stressed rats than in control rats when they were exposed to an acute stressor in the form of forced swimming (Figure 2B). This result suggests that rats exposed to repeated restraint stress and that were injected with vehicle had a more exacerbated response of the HPA axis to a new acute stressor than the control rats treated with vehicle. Interestingly, the treatment with reboxetine had an anti-stress effect on the animals, preventing the decrease in weight gain and the increase in corticosterone levels. These results, which are comparable to those of earlier studies, show that the chronic stress protocol was effective with the vehicle-treated rats (Tafet and Bernardini, 2003; Ferraz et al., 2011).

Having established that our chronic stress protocol was effective, we evaluated the effects of reboxetine on auditory attention and NE concentrations in A1. An unexpected result was that vehicle-treated control rats and vehicle-treated stressed rats (restraint) had similar performances in the attentional task (Figure 3). This result suggests that repeated injections with vehicle were also stressful for control animals and deteriorated their auditory attention, although vehicle-treated rats exposed to restraint stress had lower body weight and higher corticosterone levels than vehicle-treated control rats (Figures 2A,B). Figure 2B shows that vehicle-injected control rats exposed to acute swimming had a higher, but not statistically significant, increase in the corticosterone levels than those who were left undisturbed (acute swimming: 250.3 ± 12.6 ng/ml; unstimulated: 160.5 ± 20.2 ng/ml). If we compare this result with the ones in our previous article, in relation to the effect of acute swim on corticosterone plasma levels of control rats, never injected with vehicle, control rats exposed to acute swim did not show significant higher corticosterone levels compared to unstimulated rats (acute swim: 285.1 ± 19.9 ng/ml; unstimulated: 130.6 ± 17.1 ng/ml; Pérez et al., 2013). It is possible that injections with vehicle had been stressful for the rats at the beginning of daily injections, but after 21 days their HPA axis did adapt to vehicle injections. To support, 10 days of vehicle injection increases plasma corticosterone levels in rats (Mitra and Sapolsky, 2008) and 3 weeks of vehicle injections decreases intersections of apical dendrites of pyramidal neurons located in the medial prefrontal cortex (mPFC; Wellman, 2001), a brain area that regulated attention in rodents (Holmes and Wellman, 2009). These evidences suggest that the mPFC is more sensitive to the stressful effects induced by vehicle injections than HPA axis and the long-term effects on mPFC last until 21 days of injections. In this regard, it has been shown that chronic mild stress induces dendritic atrophy and decreases synaptic transmission in the mPFC (Brown et al., 2005; Negrón-Oyarzo et al., 2015). Therefore, in our experiments, repeated injections with vehicle may have been stressful for control rats and may have deteriorated their mPFC, impairing auditory attention as shown in Figure 3A.

On the other hand, rats that were exposed to vehicle injections and subjected to repeated restraint stress had significantly higher corticosterone levels than rats that were not disturbed. This result suggests that repeated restraint stress was more stressful for the rats than daily injections and the HPA axis of restraint-treated rats did not adapt to restraint and exacerbated their HPA axis response to a new stressor. In this context of chronic stress, we have reported that A1 shows dendritic atrophy and decreases of GABAergic efficacy, a brain area that also regulates auditory attention (Bose et al., 2010; Pérez et al., 2013). Therefore, it is possible that in our experiments vehicle injections have been less stressful than restraint, and the effects of injections on auditory attention have been linked to the mPFC and other neurotransmitter system (e.g., cholinergic system), because reboxetine treatment improved attention only in the last five trials of attentional task (Figure 3A), while repeated restraint was a strong stressor for the rats and more related to lower levels of NE in A1. These different levels of chronic stress should have different consequences in brain circuits, e.g., a more severe chronic stress could affect a longer extension of altered brain circuits (A1, subcortical nuclei and mPFC), while lower levels of chronic stress could affect a more reduced brain area (mPFC). Consequently, we propose, that reboxetine improved earlier auditory attention in animals exposed to stronger chronic stress (restrain) than in those exposed to vehicle injections (Figure 3B).

An alternative hypothesis is that the observed effects along trials in vehicle-treated animals were due to sustained attention. It is known that NE brain levels are important for maintaining sustained attention in time, and it has been shown that low dose of nortryptiline (NE reuptake blocker) can improve the performance of rats in an auditory sustained attention tasks (Roychowdhury et al., 2012). In this line, the additional effect of repeated restraint stress could be unmasked by reboxetine (Figure 3), which might diminish the impairment in performance produced by sustained attention. In addition, the effect of restraint-induced chronic stress on auditory attention depended more on NE levels in the brain than on injections-induced chronic stress. In this regard, restraint and injections can induce chronic stress in rats but both appear to be different stressors for the brain. Figure 4 shows that restraint-induced chronic stress decreased the NE levels in A1 compared to control rats that were injected with vehicle, while at behavioral level both animal groups had a similar performance in the attentional task (Figure 3).

The effects of reboxetine did not correlate with alterations in locomotor activity because reboxetine and restraint stress did not affect the distance traveled in the open field task (Figure 1B), demonstrating that reboxetine specifically affects auditory attention.

An interesting fact was that the treatment with reboxetine had a different effect on each of the control and stress experimental groups, not only at a behavioral level, but also on the NE concentration in A1. In rats that were exposed to repeated restraint stress, vehicle- and reboxetine-treated stressed rats showed similar levels of attention in the first 20 trials of the 2-ACT test, after which reboxetine-treated rats had higher attention levels and NE concentration in A1 than those vehicle-treated rats (Figures 3B, 4), indicating that NE levels were necessary to maintain the auditory attention of the animals over time during the 50 2-ACT trials. On the other hand, reboxetine treatment improved auditory attention of vehicle-treated control rats, but only in the last five trials of 2-ACT, while NE levels in A1 were similar to those of control animals treated with vehicle or reboxetine (Figure 4). If injections would have masked the effect induced by restraint, then reboxetine effects on attention would have been similar in control and restrained rats. However, results from Figures 3, 4 suggest that the effects induced by restraint on attention were more related with NE levels in the brain, while the effects related to the stress-induced by daily injections possibly were more related to changes in adrenoreceptors’ expression at neocortex.

**Scheme 2:**
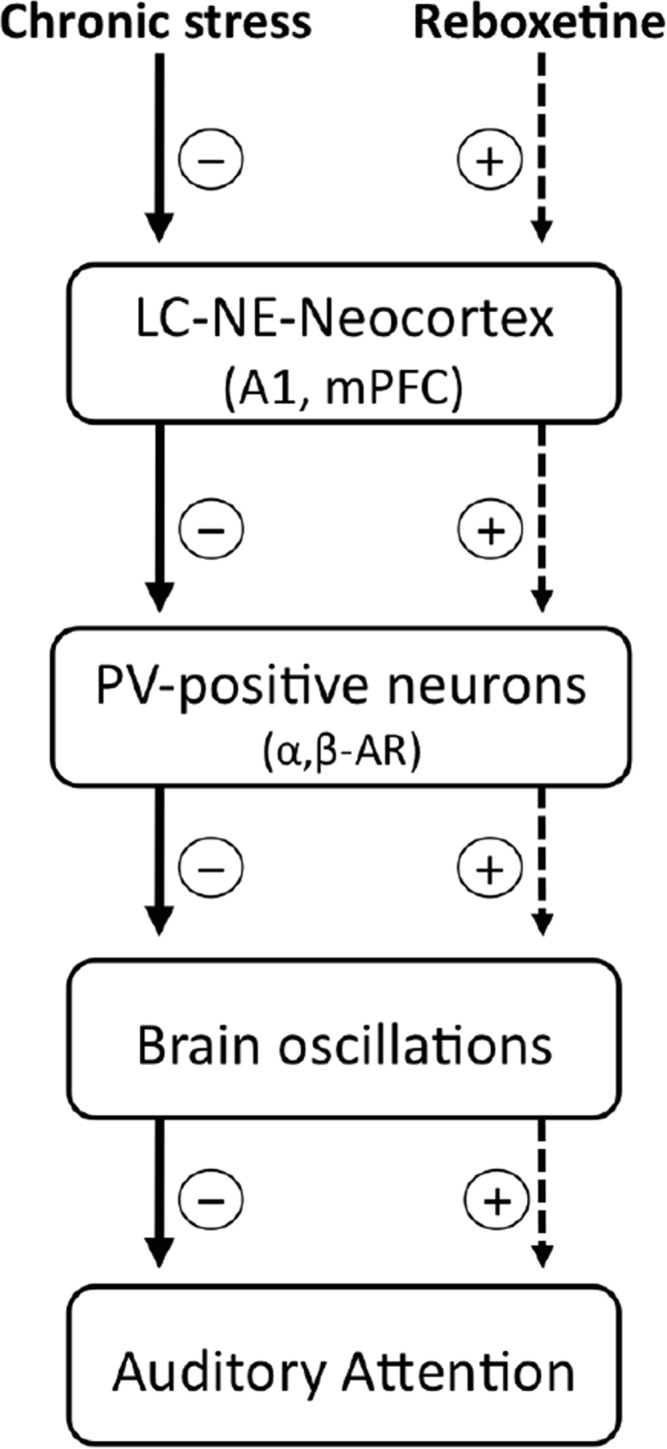
**Possible cellular mechanisms underlying the effects of chronic stress and reboxetine on auditory attention in rats.** Chronic stress reduces the activity of *locus coeruleus* and norepinephrine system (LC-NE), as a result, NE content decreases in neocortical areas which modulate attention, e.g., the primary auditory cortex (A1) and the medial prefrontal cortex (mPFC). In this context, lower levels of NE and/or changes in the expression of α and β adrenoreceptors (α, β-AR) located at interneurons could affect GABAergic activity which is crucial to generate brain oscillations in the neocortex, which in turn impairs auditory attention. On the other hand, reboxetine treatment improves these alterations.

With the results obtained in this research we cannot explain how NE affected in particular the auditory attention, so that further experiments are necessary. Despite this, we can speculate a possible mechanism and suggest it in this discussion (Scheme 2). It has been shown that an inhibition/excitation balance in A1 regulates the rat’s performance in the auditory discrimination task 2-ACT (Buzsaki and Chrobak, 1995; Cobb et al., 1995; Wehr and Zador, 2005; Tan and Wehr, 2009; Znamenskiy and Zador, 2013; Xiong et al., 2015). In this regard, chronic stress induces both atrophy of the basilar dendrites of pyramidal neurons (Bose et al., 2010) and decreases GABAergic synaptic efficacy in A1 (Pérez et al., 2013), which in turn, could modify the threshold of synaptic plasticity and induce an imbalance between the excitatory and inhibitory systems. As a result, the sustained auditory attention of the chronically stressed rats decreased than control rats in the auditory task (Pérez et al., 2013).

Interneurons exert a strong control over balance and synchronization of the brain circuit (Buzsaki and Chrobak, 1995). Therefore, modulation of GABAergic synaptic efficacy in A1 could be a main regulatory element for auditory attention. In our experiments, the effects of NE on auditory attention can be understood by considering how it modulates GABAergic neurotransmission in A1. It has been shown that NE regulates GABAergic activity by adrenoreceptors located in neurons containing the GABAergic marker parvalbumin (PV-positive neurons; Salgado et al., 2011a,b). Thus, chronic stress induced by repeated injections or restraint stress could up-regulate the expression of α1 adrenoreceptors in PV-positive neurons and as a result GABAergic activity decrease in A1 (Salgado et al., 2011a). In this regard, PV-positive neurons are crucial to generate some brain oscillations that modulate attention, for example, alpha oscillations in the A1 (Lakatos et al., 2016), selectively increase perisomatic inhibition. On the other hand, reboxetine could have increased the expression of the β and/or α2 adrenoreceptors in the PV-positive neurons. A speculative explanation is that NE could have increased GABAergic transmission in chronically stressed rats (Sessler et al., 1995; Salgado et al., 2011b, 2012; Brown et al., 2012), improving their attention in the last trials of the auditory task, as shown in Figure 3A.

On the other hand, it is possible that the effects of chronic stress and reboxetine on auditory attention could be in part induced indirectly. For example, the mPFC plays a key role in attention and NE modulates their neuronal activity (Dalley et al., 2004; Bondi et al., 2010). Therefore, in our experiments the effects of chronic stress and reboxetine on auditory attention could be generated by the effects of both, chronic stress and reboxetine, directly on mPFC. In addition, the medial geniculate nucleus (MGN, auditory thalamus) is also target of NE and MGN modulates the activity of A1 (Pannese, 2007; Tully et al., 2007) and thereby chronic stress and reboxetine also can regulate the MGN activity, which in turn could affect the auditory attention. In this regard, it is possible that chronic stress influences attention via an additional mechanism to the NE system. The auditory pathway and mPFC are strongly regulated by cholinergic system to support sustained attention (Young et al., 2004; Schofield et al., 2011; Roychowdhury et al., 2012; Poorthuis and Mansvelder, 2013; Bloem et al., 2014). It has been reported that A1, MGN, and mPFC receive cholinergic innervation from the basal forebrain and acetylcholine modulates the neuronal activity of these brain areas during attention (Passetti et al., 2000; Kamke et al., 2005; Schofield et al., 2011; Bloem et al., 2014). Moreover, behavioral studies in mice suggest that cholinergic nicotine activation is relevant for maintaining sustained attention during 5-choice serial reaction time task (Young et al., 2004).

Taken together, these evidences suggest that in our experiments NE could modulate the cholinergic system in the mPFC and/or A1, which in turn affect sustained auditory attention. For example, chronic stress could decrease NE and cholinergic activities, while reboxetine increase both NE and cholinergic activities improving sustained auditory attention as shown in Figure 3B.

In conclusion, our results suggest that chronic stress decrease the NE concentration in A1. On the other hand, reboxetine improved auditory attention and increased NE in A1 of chronically stressed rats. In addition, reboxetine had a strong anti-stress effect. We propose that NE plays a key role in the stress-induced inhibition of GABA release in A1 and by this way affects auditory attention of stressed rats.

## Author Contributions

AD-S designed research; CP-V and MFG-P performed research; CP-V, MFG-P, RS-Z and PHD analyzed data. AD-S wrote the article.

## Funding

This study was primarily funded by Anillo de Ciencia y Tecnología N° ACT1403 grant, Programa PIA of Comisión Nacional de Investigación Científica y Tecnológica (CONICYT; AD-S). We also received funding from FONDECYT 111-21205 (RS-Z).

## Conflict of Interest Statement

The authors declare that the research was conducted in the absence of any commercial or financial relationships that could be construed as a potential conflict of interest.

## Abbreviations

μL: microlitres
2-ACT: two-alternative choice task
5-HIAA: 5-Hydroxyindoleacetic acid
5-HT: serotonin
A1: primary auditory cortex
CH_3_CN: acetonitrile
cm: centimeters
CRF: corticotropin-releasing factor
dB: decibel
DS: difference score
DS-CT: difference score of correct trials
EDTA: Ethylenediaminetetraacetic acid
g: grams
GABA: gamma-Aminobutyric acid
h: hour
HPA: hypothalamus-pituitary-adrenal
HPLC: high performance liquid chromatographer
IP: Internet Protocol address
IPSCs: inhibitory postsynaptic currents
kg: kilograms
kHz: kilohertz
LC: *locus coeruleus*
m: meter
M: molar
mg: milligrams
min: minutes
mL: milliliters
mM: millimolar
mV: millivolts
μL: microlitres
N: normal
nA: nanoampere
NaH_2_PO_4_: Sodium phosphate monobasic
NE: norepinephrine
ng: nanograms
nm: nanometers
pg: picograms
s: seconds.
